# Integrated mRNA and miRNA profile expression in livers of Jinhua and Landrace pigs

**DOI:** 10.5713/ajas.18.0807

**Published:** 2019-03-07

**Authors:** Minjie Huang, Lixing Chen, Yifei Shen, Jiucheng Chen, Xiaoling Guo, Ningying Xu

**Affiliations:** 1 Department of Animal Genetics and Breeding, College of Animal Science, Zhejiang University, Hangzhou 310058, China

**Keywords:** mRNA, miRNA, Liver, Pig, RNA-seq

## Abstract

**Objective:**

To explore the molecular mechanisms of fat metabolism and deposition in pigs, an experiment was conducted to identify hepatic mRNAs and miRNAs expression and determine the potential interaction of them in two phenotypically extreme pig breeds.

**Methods:**

mRNA and miRNA profiling of liver from 70-day Jinhua (JH) and Landrace (LD) pigs were performed using RNA sequencing. Blood samples were taken to detect results of serum biochemistry. Bioinformatics analysis were applied to construct differentially expressed miRNA-mRNA network.

**Results:**

Serum total triiodothyronine and total thyroxine were significantly lower in Jinhua pigs, but the content of serum total cholesterol (TCH) and low-density lipoprotein cholesterol were strikingly higher. A total of 467 differentially expressed genes (DEGs) and 35 differentially expressed miRNAs (DE miRNAs) were identified between JH and LD groups. Gene ontology analysis suggested that DEGs were involved in oxidation-reduction, lipid biosynthetic and lipid metabolism process. Interaction network of DEGs and DE miRNAs were constructed, according to target prediction results.

**Conclusion:**

We generated transcriptome and miRNAome profiles of liver from JH and LD pig breeds which represent distinguishing phenotypes of growth and metabolism. The potential miRNA-mRNA interaction networks may provide a comprehensive understanding in the mechanism of lipid metabolism. These results serve as a basis for further investigation on biological functions of miRNAs in the porcine liver.

## INTRODUCTION

The pig is an important livestock animal and a major meat producer in China. At the same time, pigs are one of the ideal animal models for human medical research, because of high similarity in structure of physiological, anatomical, physiological and biochemical index, food structure and drug metabolism with human [1]. The Jinhua (JH) pig is a famous Chinese indigenous pig breed, being known for its slow growth and high fat deposition [2] and is widely used for ham production. On the contrary, the Landrace (LD) pig represents the fast growing lean type selected for high carcass yield [3]. Therefore, divergent performance makes the two pig breeds ideal comparisons to investigate genetic diversity and molecular mechanisms in the metabolic processes in the liver.

Liver is a critical organ in the metabolism of carbohydrates, fatty acids, amino acids and so on. It undertakes most of the synthesis, decomposition, transformation, excretion and other metabolic processes. MicroRNAs (miRNAs) are important regulators in gene expression which play key roles in biological process such as cell proliferation and differentiation [4], and pathogenesis and disease prevention [5]. The regulation of miR-122 has been associated with lipid metabolism and liver diseases [6,7]. Mir-224-5p directly targets to early growth response 2 (EGR2) and acyl-CoA synthetase long-chain family member (ACSL4), which is a novel negative regulator of adipocyte differentiation through post-transcription regulation of EGR2 during early adipogenesis and regulates fatty acid metabolism at terminal differentiation through ACSL4 [8]. MiR-130 and miR-27 suppress adipogenesis by inhibiting peroxisome proliferator-activated receptor γ (PPARγ) expression [9,10]. Although the regulatory mechanisms of metabolism in other breeds have been studied extensively, the differences between JH and LD pigs remain unreported.

The intention of this study was to characterize and compared the transcriptome profiles and of the liver in two porcine breeds with distinct phenotypes using RNA-seq technology. This study aimed to construct mRNA-miRNA regulatory network related in breedspecific growth and fat deposition.

## MATERIALS AND METHODS

### Ethics statement

The experiment was approved by the Animal Care and Use Committee of Zhejiang University, Zhejiang, China (ZJU 20160346). All procedures complied with the guidelines and the regulations for the Administration of Affairs Concerning Experimental Animals.

### Sample collection and RNA extraction

The liver tissue samples were collected from three uncastrated male JH pigs and three uncastrated male LD pigs respectively at 70-day. The liver samples were immediately frozen in liquid nitrogen and stored at −80°C until RNA extraction. Total RNA was isolated from liver tissue samples using TRIzolTM reagent (Invitrogen, Carlsbad, CA, USA), according to the manufacturer’s protocol. RNA quantity and purity were analyzed with the ND-1000 Nanodrop (Thermo Fisher, Wilmington, DE, USA). The criteria used to select the RNA for following analysis were A260/A280≥1.8, A260/A230≥2.0 and RNA integrity number >7.0.

### Serum biochemical parameters assays

The blood samples were collected from the precaval vein of each animal, kept at 37°C for 2 h, and then centrifuged at 4°C for 10 min (3,000×g). Serum glucose, triglyceride (TG), total triiodothyronine (TT3), total thyroxine (TT4), serum total cholesterol (TCH), high density lipoprotein cholesterol, low density lipoprotein cholesterol (LDLC) were determined by Affiliated Hospital of Hangzhou Normal University.

### Small RNA sequencing and data analysis

Approximately 1 μg RNA of total RNA were used to prepare a small RNA library according to instructions of NEBNext Multiplex Small RNA Library Prep Set for Illumina (Illumina, San Diego, CA, USA). The purified library products were evaluated using the Agilent 2200 TapeStation and diluted to 10 pM for cluster generation *in situ* on the HiSeq2500 single-end flow cell followed by sequencing (1×50 bp) on HiSeq 2500. After removing adapter sequences, reads containing poly-N and low quality reads, all clean reads were mapped to Rfam12.1 (rfam.xfam.org), pirnabank (pirnabank.ibab.ac.in), miRBase 21 (www.miRBase.org) and Mireap to annotate rRNA, tRNA, snRNA, snoRNA, piRNA, mature miRNAs and porcine novel miRNA. The miRNA expression levels were calculated by reads per million (RPM) values (RPM = [number of reads mapping to miRNA/number of reads in Clean data]×10^6^). Differential expression analysis between two breeds was determined by edger v3.10.0 and |log2 (fold change)| >1 and p<0.05 were set as the threshold for significance. Small RNA-seq data have been deposited in the gene expression omnibus (GEO) database and are available through the series accession numbers GSE124484.

### RNA sequencing and data analysis

Approximately 1 μg RNA per sample were used to construct the complementary (cDNA) library with NEBNext Ultra RNA Library Prep Kit for Illumina (NEB, Ipswich, MA, USA) according to the manufacturer’s instructions. After cluster generation using TruSeq Rapid SR Cluster Kit V2 (Illumina, USA), six libraries were sequenced on Illumina Hiseq 2500 platform and 50 bp single reads were generated. After filtering out adaptor sequences and removing low quality reads from raw data, the clean reads were aligned to the reference genome (*Sus scrofa* 10.2) using Tophat v2.0.13. Gene expression level was calculated by reads per kilobase per million reads (RPKM) and the numbers of reads mapped to each gene were counted by gfold v1.1.2. DEGSeq R package (1.18.0) was applied to determine differentially expressed genes (DEGs) and |log2 (fold change)| >1 and q<0.05 were set as the threshold for significance. RNA-seq data have been deposited in the GEO database and are available through the series accession numbers GSE124484.

### Real-time quantitative polymerase chain reaction validation of miRNA and mRNA

Real-time polymerase chain reaction (PCR) was performed on an ABI Step One Plus system (Applied Biosystem, Carlsbad, CA, USA) using SYBR Premix Ex Taq kit (TaKaRa, Dalian, China) with specific primers (Supplementary Table S1). Glyceraldehyde3phosphate dehydrogenase and met-tRNA were chosen as a control of mRNA and miRNA, respectively. Three biological replicates were used for each of the miRNAs and mRNAs. The method of 2^−ΔΔCt^ was used to calculate fold changes of miRNA and mRNA expression.

### Bioinformatics analysis

Gene ontology (GO) enrichment and Kyoto encyclopedia of genes and genomes (KEGG) pathway analysis for significantly differential expression were performed using the KO-Based Annotation System (KOBAS) v2.0, considering with corrected p-value <0.05 as significantly enriched. Potential targets of differentially expressed miRNAs (DE miRNAs) were predicted by PITA (http://genie.weizmann.ac.il/pubs/mir07/mir07_dyn_data.html), TargetScan (http://www.targetscan.org/) and miRanda algorithms (http://www.microrna.org/).

## RESULTS

### Metabolic characteristics of the two porcine breeds

As shown in Table 1, body weight and liver weight of LD pigs were significantly higher than those in JH pigs (p<0.05), however the liver index remained unchanged (p>0.05). The levels of TT4 and TT3 were significantly higher in LD pigs (p<0.05). Serum TCH and LDLC in Jinhua pigs were significantly higher than those in LD pigs (p<0.05), while high density lipoprotein showed no difference between two breeds. Serum glucose and serum TG did not differ between the two pig breeds, while serum insulin levels were significantly low in Jinhua pigs.

### Hepatic miRNA expression patterns of two breeds

After filtering low-quality reads and adaptor sequences, more than 98.96% of clean reads were mapped to reference genome, and more than 79.4% of mappable reads that aligned to unique miRNAs in miRbase (Supplementary Table S2). The highest expressed miRNAs by deep sequencing were ssc-mir-148a-3p, ssc-mir-122, ssc-mir-192, ssc-mir-101, ssc-mir-26a, ssc-mir-143-3p, ssc-mir-21, ssc-let-7f and ssc-let-7g in two pig breeds. We identified 35 DE miRNAs (p<0.05) in JH and LD pigs (Supplementary Figure S1, Supplementary Table S3). Compared to JH, LD demonstrated 27 up-regulated and 8 down-regulated miRNAs by calculating the log2 ratio with |log2 (fold change)| >1 and p<0.05. Among the up-regulated miRNAs, ssc-miR-199b-3p has the highest expression level, while in the down-regulated miRNAs, ssc-miR-425-3p has the highest expression level. Some of the abundant miRNAs such as ssc-miR-204, ssc-miR-145-5p, ssc-miR-199b3p, ssc-miR-199a-3p were differentially expressed (p<0.01) between the two breeds.

### Hepatic mRNA expression patterns of two breeds in porcine liver tissue

A total of six libraries JH1, JH2, JH3, LD1, LD2, and LD3 were constructed to identify the mRNA expression profiles in JH and LD pigs. As shown in Supplementary Table S4, 10575778, 13534880, 14418516, 12510741, 14427239, 14176304 raw reads were found in the six libraries. After removing low-quality reads and adaptor sequences, 10336505, 13257423, 14152632, 12279562, 14087098, 13845560 clean reads were retained for further analysis, respectively. More than 86% of clean reads were mapped to the reference genome (*Sus scrofa* 10.2). In addition, 82.08%, 87.32%, 81.28%, 83.30%, 82.18%, and 80.98% of clean reads were mapped to the exonic region (Supplementary Table S4). In total, 16,051 genes were found to be expressed in the liver of two pig breeds which calculated by RPKM and counted by gfold v1.1.2.

Differentially-expressed genes in the liver tissues of LD (control group) and JH pig breeds were acquired by DEGseq. There were 172 genes significantly upregulated by comparing the JH samples with the LD samples, whereas 295 genes were significantly down regulated (|log2(fold change)| >1 and q<0.05). The volcano map displayed DEGs (Supplementary Figure S2) significantly differentially expressed in the two breeds.

### Combined expression analysis of differentially expressed genes and miRNAs

**Figure 1:**
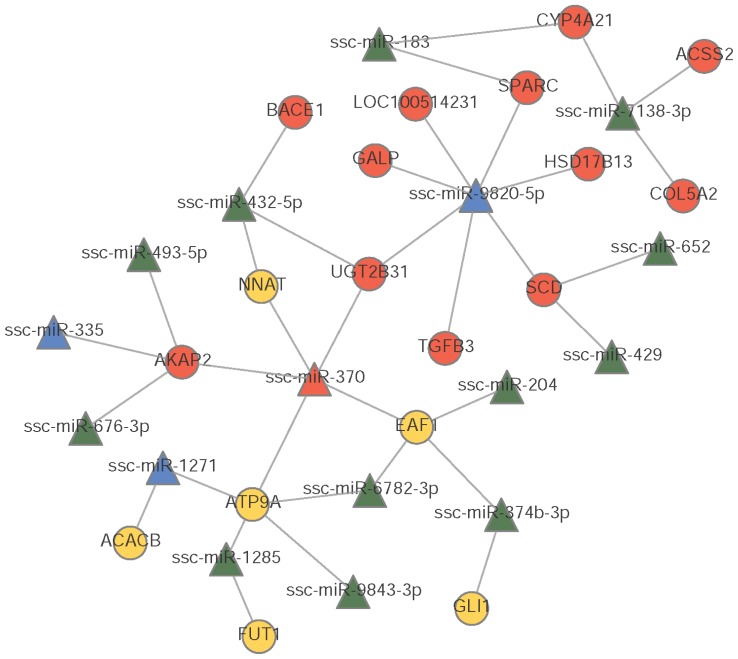
Interaction networks of differentially expressed miRNAs (DE miRNAs) to differentially genes between Jinhua and Landrace pigs. Yellow circles indicate up-regulated genes. Red circles indicate down-regulated genes. Blue triangles indicate up-regulated miRNAs. Green triangles indicate down-regulated miRNAs. Straight lines indicate interaction relationships.

In this study, the target genes were predicted by PITA, TargetScan and miRanda algorithms. The 8 down-regulated and 27 up-regulated miRNAs (p<0.05) were associated with 881 gene targets. Among 881 genes targeted by significantly DE miRNAs, 705 were expressed in the two pig breeds. For the two breeds, there were 28 DEGs (|log2 (fold change)| >1 and q<0.05) regulated by 25 DE miRNAs. The potential regulation network of DE miRNAs and DEGs is shown in Figure 1.

### Functional enrichment of differentially expressed genes

**Figure 2:**
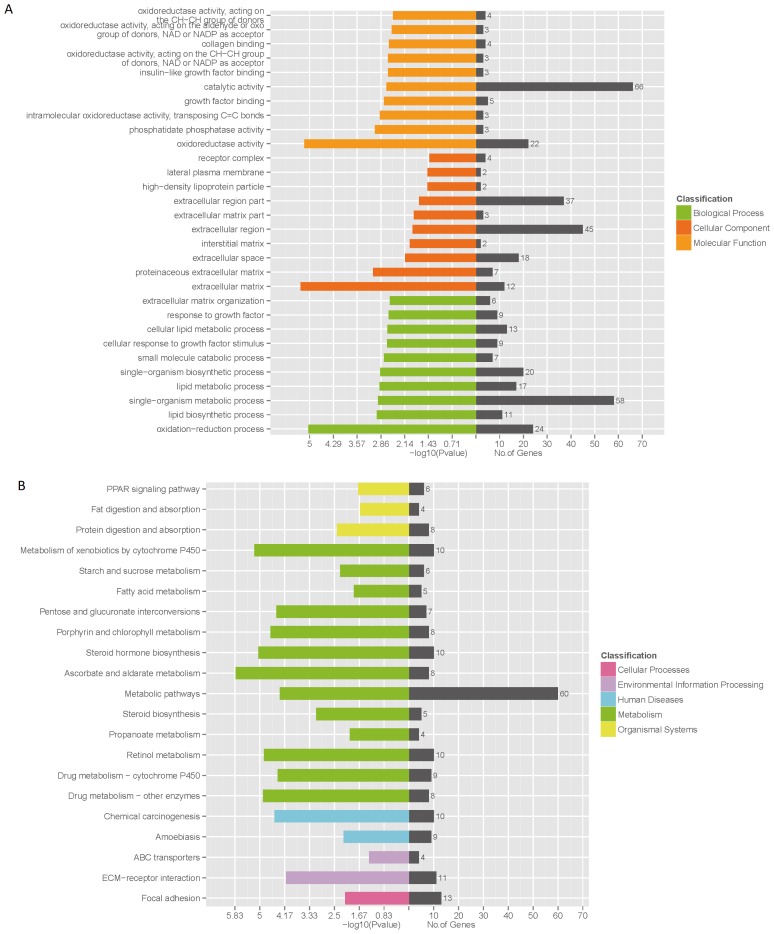
Bioinformatics analysis of differentially expressed genes (DEGs) by KOBAS 2.0. (A) Gene ontology enrichment analysis of DEGs and (B) Kyoto encyclopedia of genes and genomes pathway analysis of DEGs.

The GO enrichment analysis was applied to the DEGs to explore the biological function between two pig breeds (Figure 2A). The enriched GO targets were mainly associated with oxidation-reduction, lipid biosynthetic and lipid metabolic process. The DEGs were classified according to KEGG function annotations to identify the pathways (Figure 2B). The involved pathways included: metabolic pathways, fatty acid metabolism, PPAR signaling pathway, fat digestion and absorption and protein digestion and absorption.

### Quantitative real-time polymerase chain reaction validation for miRNAs and mRNAs

**Figure 3:**
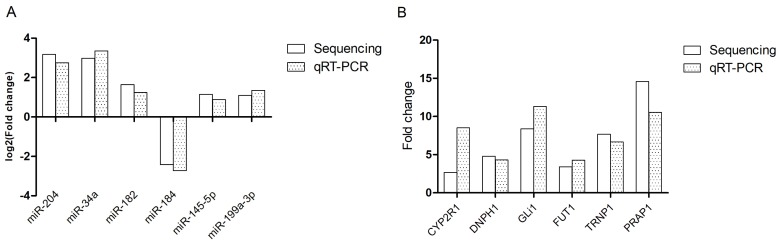
Validation of the RNA-seq data by quantitative real-time polymerase chain reaction. Six miRNAs (A) and six mRNAs (B) were randomly selected from each analysis group with three biological replicates.

To validate reliability of RNA-seq results, six DE miRNAs and six mRNAs (miR-204, miR-34a, miR-182, miR-184, miR-145-5p, miR-199a-3p, cytochrome P450, family 2, subfamily R, polypeptide 1 (CYP2R1), 2′-deoxynucleoside 5′-phosphate N-hydrolase 1, GLI family zinc finger 1 (GLi1), fucosyltransferase 1, TMF1 regulated nuclear protein 1, proline rich acidic protein 1 were randomly selected and subjected to quantitative real-time polymerase chain reaction (qRT-PCR) (Figure 3). The results indicated that the DE miRNAs and mRNAs with RNA-seq were reliable. As shown in Figure 3, CYP2R1 didn’t show consistent expression between RNA-seq data and qRT-PCR data. It was probably caused by physiological differences between samples or sensitivity of different methods.

## DISCUSSION

The liver is important to vertebrates and some other animals as it plays a major role in carbohydrate, protein, amino acid and lipid metabolism [11]. In this study, significant differences in physiological and biochemical traits were observed between growing LD and JH pigs. The body weight and liver weight indicated discrepancy between cell proliferation and differentiation in two breeds. The levels of serum TT3 and TT4 were significantly higher in LD pigs, which was in agreement with study of Jinhua pigs and Yorkshire pigs [12]. TT3 and TT4 play a role in accelerating the decomposition of fat in adipose tissue and increase the content of free fatty acids in blood [13] which also enhance the function of growth hormone to activate the adenylate cyclase in order to increase steatolysis through cAMP-protein kinase system [14]. The results showed that the content of serum TCH and LDLC were strikingly higher in Jinhua pigs which indicated higher rate of cholesterol metabolism. What’s more, leptin produced by adipose tissue participates in the metabolism of lipid in the liver which influences the synthesis of phosphoenolpyruvate carboxylase and gluconeogenesis in order to restrict the synthesis of TG and to enhance the sensitivity of insulin to the liver [15]. We identified 467 DEGs between JH and LD pig breeds. Various of them were associated with lipid metabolic and biosynthetic processes (Figure 2A). There were 6 DEGs involved in PPAR signal pathway through KEGG pathway analysis. Among them, acyl-CoA dehydrogenase long chain and solute carrier family 27 member 4 had higher expression in JH liver which were essential for oxidizing unsaturated fatty acids [16] and transporting fatty acids across the membrane [17]. The higher expression of apolipo protein A4 indicated antioxidant properties and reducing LDLC peroxidation [18], which may respond to serum LDLC increased significantly in JH pigs. Differences in biochemical performances and gene expression profiles showed the comprehensive scope of the liver metabolism processes varied between the two breeds.

MiRNAs play important roles in the post-transcription regulation which is involved in various developmental and physiological processes [19]. In our study we found out that several DE miRNAs were related to fat metabolism. Among them, miR-374b directly targets CCAAT enhancer binding protein beta which is an important liver transcription factor involved in the effect of maternal dietary protein on offspring lipid metabolism in Meishan pigs [20]. MiR-370 can control expression of miR-122 and carnitine palmitoyltransferase 1A and then affects lipid metabolism [21]. Inhibition of miR-145 which was abundant expressed in LD pigs regulates ATP binding cassette subfamily A member 1 expression and promotes high-density lipoprotein biogenesis in the liver [22]. MiR-34a acts an inhibitor of beige and brown fat formation by suppressing adipocyte fibroblast growth factor 21 signaling and sirtuin 1 function [23] and our observations indicated that miR-34a was up-regulated in the LD group.

MiRNA-mRNA co-regulatory networks have provided a comprehensive result for understanding the mechanism of lipid metabolism. In the present study, despite the fact that the differences in growth and fat deposition are significant between the two pig breeds, there are still no effective networks research on it. After filtering the target genes predicted by bioinformatics software which differentially expressed in the two pig breeds, miRNA-mRNA potential networks were constructed for more understanding. As shown in the regulatory network (Figure 1), MiR-204 which was down-regulated in JH pigs was reported to be up-regulated during adipogenesis of human bone marrow stem cells and favors adipogenesis when overexpressed [24]. ELL associated factor 1 (EAF1), the predicted target of miR-204 which is expressed higher in JH pigs, negatively regulates canonical Wnt/β-catenin signaling [25] involved in porcine adipose tissue development [26]. The GLi1 is a transcription factor of the Hedgehog (Hh) signaling pathway which inhibits fat accumulation in drosophila and mammalian models [27]. Acetyl-CoA carboxylase beta plays an important role in fatty acids oxidation [28]. What’s more, stearoyl-CoA desaturase (SCD) is related to intramuscular fat deposition through synthesis and desaturation of fatty acids in pigs [29]. MiR-429 which interacted with SCD in our prediction is upregulated during adipogenic differentiation in mouse ST2 mesenchymal stem cells [30]. In our study, miR-370 expressed higher in LD group which influence on oxidation of fatty acids and synthesis of TG [21]. Our findings indicated that the differential expression of genes and miRNAs might contribute to lipid metabolism and fat deposition in pigs.

## Supplementary Data



## Figures and Tables

**Table 1:** Metabolic and endocrine parameters in the Jinhua and Landrace pig breeds.

Items	Jinhua	Landrace	p-value
Body weight (kg)	8.87±0.09	14.03±0.12	0.000
Liver weight (g)	181.27±3.23	321.13±2.56	0.000
Liver index (g/kg)	20.82±0.62	22.17±0.02	0.064
Serum insulin (mIU/L)	4.23±0.20	8.12±0.14	0.000
Serum glucose (mmol/L)	7.21±0.14	7.64±0.14	0.092
Serum total thyroxine level (nmol/L)	1.15±0.02	1.26±0.01	0.020
Serum total triiodothyronine level (nmol/L)	43.22±3.58	69.47±7.07	0.030
Serum TG (mmol/L)	0.50±0.00	0.49±0.00	0.101
Serum TCH (mmol/L)	3.46±0.04	2.49±0.10	0.001
Serum HDLC (mmol/L)	0.77±0.02	0.86±0.04	0.103
Serum LDLC (mmol/L)	1.94±0.05	1.25±0.00	0.000

TG, triglyceride; TCH, total cholesterol; HDLC, high density lipoprotein cholesterol; LDLC, low density lipoprotein cholesterol.

All data were expressed as mean±standard error of the mean and p<0.05 was considered significant.

## References

[1] Miller ER, Ullrey DE (1987). The pig as a model for human nutrition. Annu Rev Nutr.

[2] Ting W, Zhenhai Z, Zhangqin Y (2013). Distinctive genes determine different intramuscular fat and muscle fiber ratios of the *longissimus dorsi* muscles in Jinhua and landrace pigs. PLoS One.

[3] Chen P, Baas TJ, Mabry JW, Koehler KJ (2003). Genetic correlations between lean growth and litter traits in U.S. Yorkshire, Duroc, Hampshire, and Landrace pigs. J Anim Sci.

[4] Mckenna LB, Schug J, Vourekas A (2010). MicroRNAs control intestinal epithelial differentiation, architecture, and barrier function. Gastroenterology.

[5] Meng SX, Yi-Yang HU, Feng Q (2014). Application of microRNA in prevention and treatment of nonalcoholic fatty liver disease with traditional Chinese medicine. Chin J Tradit Chin Med Pharm.

[6] Christine E, Scott D, Murray SF (2006). miR-122 regulation of lipid metabolism revealed by *in vivo* antisense targeting. Cell Metab.

[7] Sharda T, Kalpana G (2015). miR-122 is a unique molecule with great potential in diagnosis, prognosis of liver disease, and therapy both as miRNA mimic and antimir. Curr Gene Ther.

[8] Peng Y, Xiang H, Chen C (2013). MiR-224 impairs adipocyte early differentiation and regulates fatty acid metabolism. Int J Biochem Cell B.

[9] Eun Kyung L, Jeong LM, Kotb A (2011). miR-130 suppresses adipogenesis by inhibiting peroxisome proliferator-activated receptor gamma expression. Mol Cell Biol.

[10] Qun L, Zhanguo G, Alarcon RM, Jianping Y, Zhong Y (2009). A role of *miR*-27 in the regulation of adipogenesis. FEBS J.

[11] Spurlock ME, Gabler NK (2008). The development of porcine models of obesity and the metabolic syndrome. J Nutr.

[12] Shen Y, Mao H, Huang M (2016). Long noncoding RNA and mRNA expression profiles in the thyroid gland of two phenotypically extreme pig breeds using ribo-zero RNA sequencing. Genes.

[13] Xuguang Z, Sheue-Yann C (2010). New insights into regulation of lipid metabolism by thyroid hormone. Curr Opin Endocrinol Diabetes Obes.

[14] Etherton TD, Louveau I, SøRensen MT, Chaudhuri S (1993). Mechanisms by which somatotropin decreases adipose tissue growth. Am J Clin Nutr.

[15] Uygun A, Kadayifci A, Yesilova Z (2000). Serum leptin levels in patients with nonalcoholic steatohepatitis. Am J Gastroenterol.

[16] Chegary M, Ht B, Ruiter JP (2009). Mitochondrial long chain fatty acid β-oxidation in man and mouse. Biochim Biophys Acta.

[17] Newberry EP, Xie Y, Kennedy SM, Luo J, Davidson NO (2006). Protection against Western diet-induced obesity and hepatic steatosis in liver fatty acid-binding protein knockout mice. Hepatology.

[18] Wong WM, Gerry AB, Putt W (2007). Common variants of apolipoprotein A-IV differ in their ability to inhibit low density lipoprotein oxidation. Atherosclerosis.

[19] Rafael K, Moeller MJ (2011). The next level of complexity: post-transcriptional regulation by microRNAs. Kidney Int.

[20] Pan S, Zheng Y, Zhao R, Yang X (2013). MicroRNA-130b and microRNA-374b mediate the effect of maternal dietary protein on offspring lipid metabolism in meishan pigs. Br J Nutr.

[21] Dimitrios I, Konstantinos D, Yaeko H, Goldberg IJ, Zannis VI (2010). MicroRNA-370 controls the expression of microRNA-122 and Cpt1alpha and affects lipid metabolism. J Lipid Res.

[22] Kang MH, Zhang LH, Wijesekara N (2013). Regulation of ABCA1 Protein Expression and Function in Hepatic and Pancreatic Islet Cells by miR-145. Arterioscler Thromb Vasc Biol.

[23] Ting F, Sunmi S, Sunge C (2014). MicroRNA 34a inhibits beige and brown fat formation in obesity in part by suppressing adipocyte fibroblast growth factor 21 signaling and SIRT1 function. Mol Cell Biol.

[24] Jian Huang LZ, Xing Lianping, Chen Di (2010). MicroRNA-204 regulates Runx2 protein expression and mesenchymal progenitor cell differentiation. Stem Cells.

[25] Jing-Xia L, Dawei Z, Xunwei X (2013). Eaf1 and Eaf2 negatively regulate canonical Wnt/β-catenin signaling. Development.

[26] Xiao L, Li H, Yang G (2008). Sequential expression of Wnt/β-catenin signal pathway related genes and adipocyte transcription factors during porcine adipose tissue development. Chin J Biotechnol.

[27] Suh JM, Gao X, Mckay J, Mckay R, Salo Z, Graff JM (2006). Hedgehog signaling plays a conserved role in inhibiting fat formation. Cell Metab.

[28] Riancho JA, Vázquez L, García-Pérez MA (2011). Association of ACACB polymorphisms with obesity and diabetes. Mol Genet Metab.

[29] Zappaterra M, Deserti M, Mazza R, Braglia S, Zambonelli P, Davoli R (2016). A gene and protein expression study on four porcine genes related to intramuscular fat deposition. Meat Sci.

[30] Yongdong P, Shulong Y, Huanan L, Hong X, Jian P, Siwen J (2014). MicroRNAs: emerging roles in adipogenesis and obesity. Cell Signal.

