# Supplementary material for: Integrated mRNA and miRNA profile expression in livers of Jinhua and Landrace pigs
Source: Asian-Australas J Anim Sci. 2019 Mar 7;32(10):1483–90. doi: 10.5713/ajas.18.0807 (PMC6718901; doi:10.5713/ajas.18.0807)
Supplement: Supplementary file 1 [file ajas-18-0807-suppl.pdf]

Table S1

Gene name	Primer sequence
Met-tRNA	CAGAGTGGCGCAGCGGAAGC
ssc-miR-204	TTCCCTTTGTCATCCTATGCCT
ssc-miR-34a	TGGCAGTGTCTTAGCTGGTTGT
ssc-miR-182	TTTGGCAATGGTAGAACTCACACT
ssc-miR-184	TGGACGGAGAACTGATAAGGGT
ssc-miR-145-5p	GTCCAGTTTTCCCAGGAATCCCTT
ssc-miR-199a-3p	ACAGTAGTCTGCACATTGGTTA
GAPDH	F:GAAGATGGTGATGGGATTTC
	R:GATGGTGAAGGTCGGAGTG
CYP2R1	F:CAATAGGATGCGGCGGAGTTG
	R:TAGCGAGTAGATGTTGCCGAT
DNPH1	F:AGTGGTTGGAGCGCTGAAG
	R:GCTCATGGATGGCTCTGTCA
GLi1	F:GAAGTCTGAGCTGGATGTGCTGGTG
	R:CATGGATGTGTTCGCTGTTGATGTG
FUT1	F:CCAACGCCTCCGATTCCTGT
	R:GTGCATGGCAGGCTGGATGA
TRNP1	F:CAAACCCGAGGATCGACTGT
	R:AAGGTCGGAGTTGAAGGTGG
PRAP1	F:AGAGCACCCTCCACACTGA
	R:GTCCAGGGTGTCCTTGTTCC

Table S2. Summary of the sequencing reads alignment to the reference genome and annotation of ncRNAs

Category	JH1	JH2	JH3	LD1	LD2	LD3
Clean reads	12632521	12199149	10672375	13341029	12713116	13932322
Mapping reads	12557637	1.21E+08	10601280	12608372	12608372	13819240
	99.41%	98.96%	99.33%	99.15%	99.18%	99.19%
miRNA	10966116	10284923	8971326	11366345	100948451	11768669
	86.81%	84.31%	84.06%	85.20%	79.40%	84.47%
tRNA	48194	80774	75276	95140	93785	55330
rRNA	450458	607666	470347	589176	879814	740077
snRNA	9790	10632	11794	10397	11081	9845
snoRNA	68116	131849	71205	87560	43406	50019
Y RNA etc	47806	41371	44770	53997	63806	64980
Others	1042041	1041934	1027657	1138414	1526379	1243402

Table S3

miRNA_ID	JH1,JH2,JH3	LD1,LD2,LD3	up/down	log2(foldcł	P-value	Significance-Lab
ssc-miR-204	143.3017	1294.7377	up	3.1755	2.11E-21	**
ssc-miR-34a	6.4556	51.1136	up	2.9851	1.26E-12	**
ssc-miR-29b	35.8188	12.1915	down	-1.5548	2.36E-08	**
ssc-miR-9843-3p	3.3733	14.9737	up	2.1502	2.73E-06	**
ssc-miR-369	0	1.603	up	7.3246	3.53E-06	**
ssc-miR-184	24.2232	4.5116	down	-2.4247	1.49E-05	**
ssc-miR-429	2.5116	10.0729	up	2.0038	7.25E-05	**
ssc-miR-6782-3p	2.1409	15.6437	up	2.8693	0.000159	**
ssc-miR-182	40.9322	127.2289	up	1.6361	0.000301	**
ssc-miR-145-5p	905.6333	2008.4624	up	1.1491	0.000303	**
ssc-miR-676-3p	19.8427	48.9872	up	1.3038	0.000324	**
ssc-miR-199b-3p	3621.8412	8020.0944	up	1.1469	0.000437	**
ssc-miR-183	3.5531	11.5173	up	1.6967	0.000792	**
ssc-miR-199a-3p	3664.866	7845.0885	up	1.098	0.000935	**
ssc-miR-7135-3p	0	2.0022	up	7.6454	0.001244	**
ssc-miR-190a	1.0776	0	down	-6.7517	0.002018	**
ssc-miR-127	46.9284	138.6024	up	1.5624	0.002307	**
ssc-miR-370	1.8371	8.2565	up	2.1681	0.002548	**
ssc-miR-425-3p	129.9634	63.3639	down	-1.0364	0.002563	**
ssc-miR-181d-5p	11.562	26.5066	up	1.197	0.002575	**
ssc-miR-374b-5p	150.12	307.3642	up	1.0338	0.002744	**
ssc-miR-432-5p	0	1.0489	up	6.7127	0.004829	**
ssc-miR-493-5p	0	1.0271	up	6.6824	0.005356	**
ssc-miR-199b-5p	425.9519	916.0519	up	1.1047	0.005816	**
ssc-miR-652	0	1	up	6.6439	0.005959	**
ssc-miR-9820-5p	0.7105	0	down	-6.1508	0.006687	**
ssc-miR-1271	0.629	0	down	-5.975	0.006713	**
ssc-miR-219a	3.1225	1.4014	down	-1.1558	0.007524	**
ssc-miR-335	5.9349	2.2906	down	-1.3735	0.009085	**
ssc-miR-181b	94.1269	189.6128	up	1.0104	0.009186	**
ssc-miR-1285	0	0.7181	up	6.1661	0.009504	**
ssc-miR-7138-3p	0	0.5972	up	5.9001	0.015403	*
ssc-miR-133a-3p	6.156	13.8854	up	1.1735	0.017159	*
ssc-miR-374b-3p	4.1827	9.1123	up	1.1234	0.045271	*
ssc-miR-146b	26.8199	54.597	up	1.0255	0.048875	*

Table S4. Characteristics of mRNA sequencing

Items	JH1	JH2	JH3	LD1	LD2	LD3
Raw reads	10,575,778.00	13,534,880.00	14,418,516.00	12,510,741.00	14,427,239.00	14,176,304.00
Clean reads	10,336,505	13,257,423	14,152,632	12,279,562	14,087,098	13,845,560
otal mappe	9,150,794	11,485,590	12,853,044	11,088,637	12,485,248	12,574,527
Q30(%)	95.08	96.06	96.25	96.28	94.88	94.94
GC content	51.08	50.04	51.53	49.93	50.73	49.64
ultiple mapp	893,447	1,117,776	1,128,657	946,685	1,242,648	1,097,450
	8.64%	8.43%	7.97%	7.71%	8.82%	7.93%
iquely mapp	8,257,347	10,367,814	11,724,387	10,141,952	11,242,600	11,477,077
	79.89%	78.20%	82.84%	82.59%	79.81%	82.89%
ads map to	4,404,010	5,553,415	6,250,831	5,381,477	5,847,533	6,213,086
	42.61%	41.89%	44.17%	43.82%	41.51%	44.87%
eads map to	4,746,784	5,932,175	6,602,213	5,707,160	6,637,715	6,361,441
	45.92%	44.75%	46.65%	46.48%	47.12%	45.95%
exonic	82.08%	87.32%	81.28%	83.30%	81.18%	80.98%
intronic	6.44%	4.90%	9.69%	5.99%	6.27%	6.56%
intergenic	11.48%	7.78%	9.03%	10.71%	12.55%	12.46%

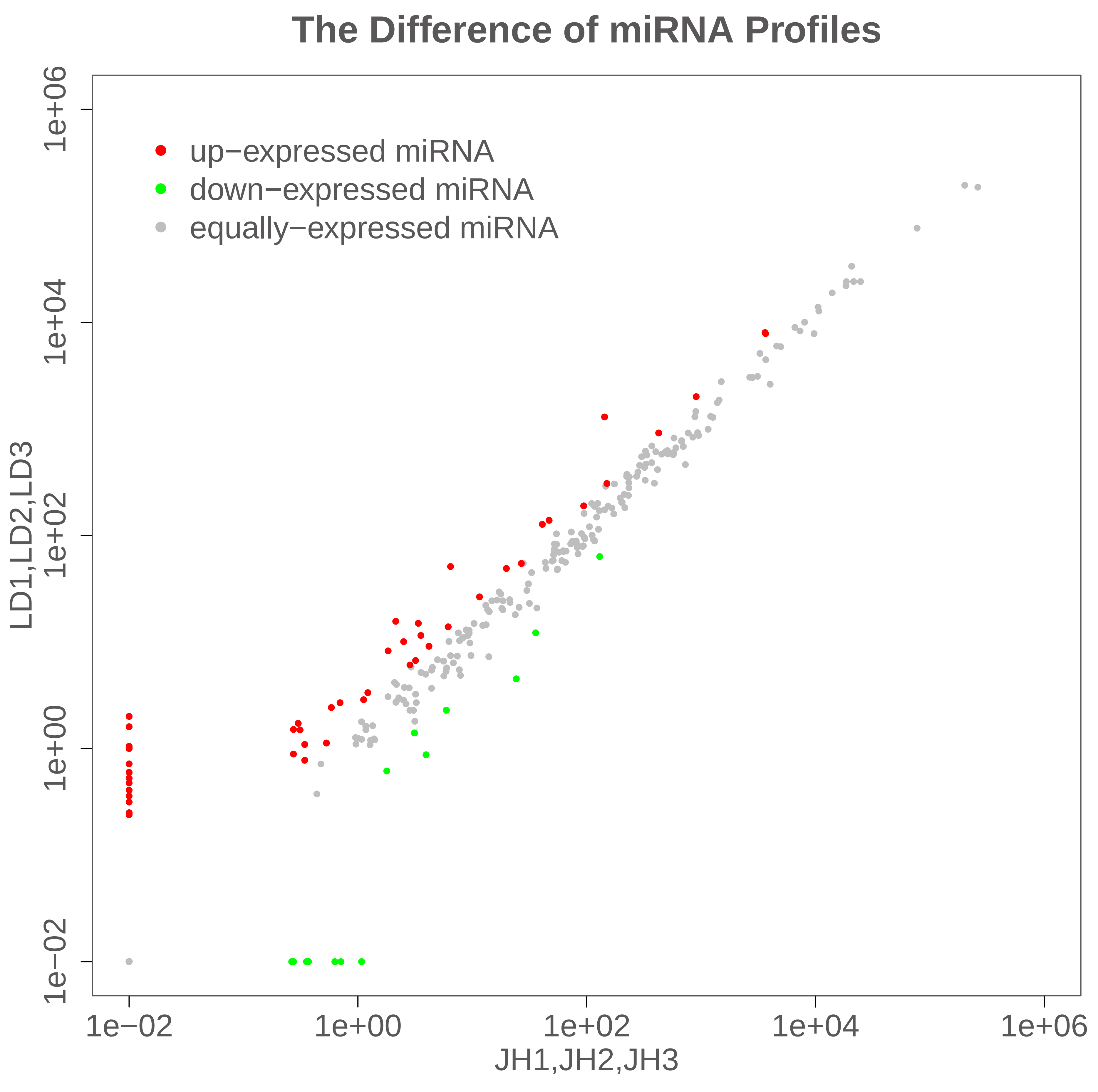

The Difference of miRNA Profiles
up−expressed miRNA
down−expressed miRNA
equally−expressed miRNA
1e+06
1e+04
1e+02
1e+00
1e−02
LD1,LD2,LD3
1e−02
1e+00
1e+02
1e+04
1e+06
JH1,JH2,JH3

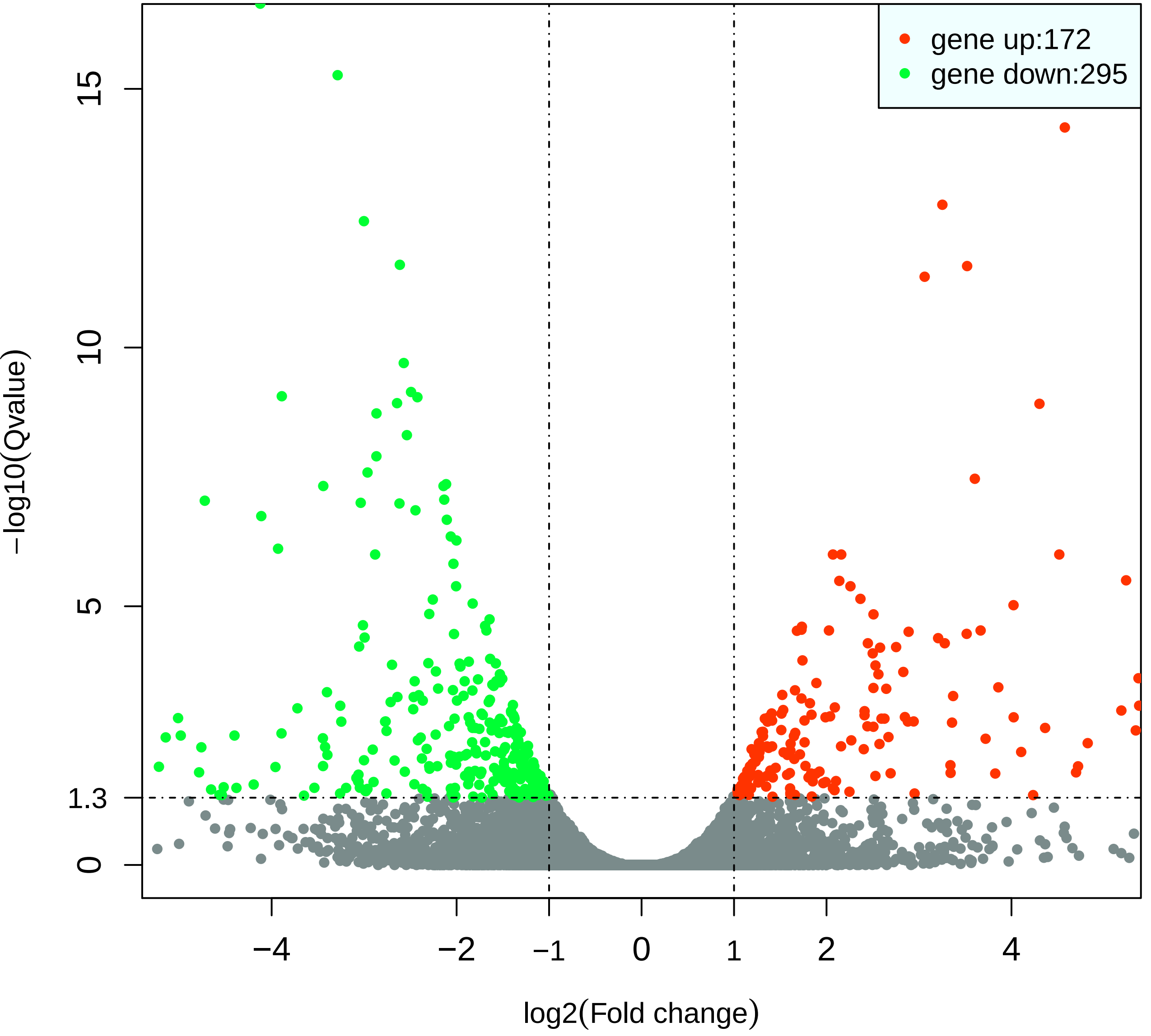
gene up:172
gene down:295
−log10(Qvalue)
15
10
5
1.3
0
−4
−2
−1
0
1
2
4
log2(Fold change)
